# An Analysis of the Early Responses to Low-Temperature Stress in Oil Palm Using Integrative Physiology and Proteomics

**DOI:** 10.3390/ijms27167418

**Published:** 2026-08-19

**Authors:** Yuxin Liu, Yuqiao Song, Jerome Jeyakumar John Martin, Shuanghong Cheng, Xiao-Yu Liu, Xinyu Li, Chengxu Sun, Wen-Rao Li, Hongxing Cao

**Affiliations:** 1 State Key Laboratory of Tropical Crop Breeding, Chinese Academy of Tropical Agricultural Sciences, Haikou 571101, China; liu2556017382@163.com (Y.L.); 17638596793@163.com (Y.S.); jeromejeyakumarj@gmail.com (J.J.J.M.); liuxy86@catas.cn (X.-Y.L.); lixinyu@catas.cn (X.L.); suncx@catas.cn (C.S.); 2 Coconut Research Institute, Chinese Academy of Tropical Agricultural Sciences, Wenchang 571339, China; 3 College of Tropical Crops, Department of Forestry, Yunnan Agricultural University, Pu’er 665000, China; 1999033@ynau.edu.cn; 4 School of Life Sciences, Henan University, Kaifeng 475004, China

**Keywords:** oil palm, low-temperature stress, physiology, proteomics

## Abstract

Oil palm (*Elaeis guineensis* Jacq.) is a quintessential tropical oilseed crop, whose distribution and yield are significantly constrained by low-temperature stress. This study conducted physiological and proteomic analyses to investigate the early response mechanisms of one-year-old thin-shelled oil palm seedlings exposed to low temperatures of 8 °C for durations of 0, 0.5, 1, 2, 4, and 8 h. The results indicated that low-temperature treatment inhibited chlorophyll accumulation, reduced photosynthetic efficiency, and increased the levels of malondialdehyde (MDA) and soluble sugars. Concurrently, the activities of antioxidant enzymes exhibited a transient increase, suggesting an imbalance in the antioxidant defense system during the later stages of stress. Proteomics results indicate that the core pathways involved are fatty acid degradation and the regulatory pathways for starch and sucrose metabolism. Within these pathways, key proteins such as ACSL (long-chain fatty acid-CoA ligase), paaF (enoyl-CoA hydratase), HADH (3-hydroxyacyl-CoA dehydrogenase), and HADHA (enoyl-CoA hydratase/long-chain 3-hydroxyacyl-CoA dehydrogenase) are identified among 13 differential accumulation protein (DAPs). It is hypothesized that these key proteins work in concert to regulate osmosis, scavenge reactive oxygen species (ROS), stabilize membranes, and supply energy. Through physiological and proteomic analyses, this study identified physiological indicators, key proteins, and regulatory pathways associated with cold tolerance in oil palm, thus providing a theoretical basis for breeding low-temperature-tolerant oil palm varieties. The findings contribute to the development of low-temperature-tolerant oil palm cultivars.

## 1. Introduction

The oil palm, scientifically known as *Elaeis guineensis Jacq.*, is often referred to as the ‘King of Tropical Oils’ [1] and is recognized as the leading source of vegetable oil worldwide [2]. This species thrives in humid environments with high temperatures, ideally ranging from 24 to 27 °C. Growth ceases when temperatures drop below 12 °C, which significantly impacts both fruit development and overall yield [3]. Therefore, it is crucial to explore the molecular mechanisms associated with low-temperature stress and to develop cultivars capable of withstanding lower temperatures, thereby enabling expansion into cooler climate regions.

Low-temperature stress represents a significant abiotic factor that restricts both the distribution and productivity of plants [4]. To counteract damage caused by temperature fluctuations, plants have developed a range of adaptive strategies, encompassing the physiological and proteomic [5,6]. On a physiological level, essential compounds such as soluble sugars, flavonoids, proline (PRO), superoxide dismutase (SOD) and peroxidase (POD) are vital for preserving membrane integrity and stability, as well as for neutralizing reactive oxygen species (ROS). For example, in *Hordeum vulgare var. nudum*, a rise in soluble sugar and PRO levels, alongside increased activity of superoxide SOD and POD, contributes to improved low-temperature stress [7]. In a similar vein, *Camellia sinensis* exhibits reduced frost damage to young shoots through the accumulation of flavonoids, especially catechin derivatives, which enhance their antioxidant potential [8].

Under low-temperature stress, plants typically reduce water and heat loss by closing their stomata, which results in an increase in intercellular CO_2_ concentration (*C_i_*) [9]. This indicates that stomatal regulation plays a crucial role in the response to low temperatures [10]. However, stomatal limitation is not the only mechanism underlying photosynthetic inhibition. Research has shown that the primary damage caused by low-temperature stress is the disruption of thylakoid membrane structure, which directly inhibits the function of the photosystem II (PSII) reaction center, impedes the electron transport chain, and reduces the activity of key enzymes [11]. Simultaneously, stomatal closure restricts CO_2_ supply, exacerbates photorepression, and leads to the accumulation of reactive oxygen species [12]. Under low-temperature conditions, chlorophyll content in leaves decreases significantly, while the net photosynthetic rate (*P_n_*), stomatal conductance (*G_s_*), and transpiration rate (*T_r_*) also decline. The maximum quantum efficiency of PSII photochemistry (*F_v_/F_m_*) and effective quantum yield of photosystem II (*ΦPSII*) of PSII are markedly suppressed, and the activity of PSI is similarly weakened [13]. Moreover, electron transport from QA to QB on the acceptor side of PSII is impeded, leading to a reduction in carboxylation efficiency (CE), which ultimately limits photosynthetic carbon assimilation capacity [13,14]. Studies on *Cucumis melo* seedlings further revealed that *P_n_* plummeted by 80.8%, even when *G_s_* was reduced by only 28.5%, thereby confirming the mechanism of photosynthetic decline driven by non-stomatal factors [15].

Proteins are the primary executors of biological activities, and changes in their expression are closely associated with phenotypic variation [16]. Therefore, analyzing the low-temperature response mechanism of oil palm at the protein level is of significant importance. Studies have demonstrated that in the model plant *Arabidopsis thaliana*, fatty acid β-oxidation serves as a pivotal pathway for the degradation of stored lipids and energy supply. This process primarily occurs in peroxisomes and plays an essential role during seed germination and seedling growth. Moreover, this pathway is not only involved in energy metabolism but is also closely related to the biosynthesis of plant hormones such as jasmonic acid [17]. Studies on *Oryza sativa* have shown that the starch and sucrose metabolic pathways are involved in the regulation of the cold response [18]. Similarly, starch and sucrose metabolism in oil palm has a decisive effect on fruit oil yield. As key metabolites closely linked to oil production, they provide important theoretical guidance for future variety breeding [19].

In recent years, significant progress has been made in understanding the mechanisms underlying the response of oil palm to low temperatures. At the physiological and biochemical levels, PRO, SOD, POD, and malondialdehyde (MDA) have been identified as key indicators for distinguishing cold-tolerant materials [20]. Regarding photosynthetic parameters, when the temperature decreases from 25 °C to 4 °C, the net *P_n_*, *G_s_*, and *T_r_* decrease significantly, while the *C_i_* declines and the stomatal limiting value (Ls) and water use efficiency (WUE) increase. This indicates that the decline in photosynthesis at this stage is primarily due to stomatal limitation. However, when the temperature is further reduced to 1 °C, *C_i_* increases while Ls decreases, suggesting that non-stomatal factors become the primary limiting factors [21]. Changes in chlorophyll fluorescence parameters further elucidate the mechanism of light inhibition. As the temperature decreases, the initial fluorescence (*F_o_*) continues to rise, while the *F_v_/F_m_*, the photochemical quenching coefficient (*qP*), and the non-photochemical quenching coefficient (*qN*) steadily decline, with these changes being particularly pronounced below 4 °C. This indicates that low temperatures lead to damage to the PSII reaction center, accumulation of excess excitation energy, and a reduction in heat dissipation capacity [22,23]. Furthermore, changes in photosynthetic mechanisms and fluorescence parameters under low-temperature, low-light conditions are more pronounced than under dark conditions, exacerbating light inhibition [23].

Metabolomic analysis has revealed significant enrichment in arginine–proline metabolism, phytohormone synthesis, and flavonoid pathways. The accumulation of key metabolites, such as citric acid and L-aspartic acid, is a defining characteristic of enhanced cold tolerance [24]. Concurrently, the development and functional annotation of 6603 SSR molecular markers have provided a powerful tool for mapping cold-tolerance genes in oil palm [25]. The aforementioned studies have preliminarily elucidated the mechanisms underlying cold-tolerance responses in oil palm. However, current research predominantly focuses on physiological indicators, metabolomics, and transcriptomics analyses, with a notable lack of in-depth studies at the proteomics level.

It is hypothesized that early-stage low-temperature stress affects protein expression in oil palm seedlings, leading to alterations in relevant physiological and regulatory pathways that subsequently influence their low-temperature tolerance. To test this hypothesis, this study utilized one-year-old thin-shelled oil palm seedlings as experimental material. By subjecting the seedlings to low-temperature treatment at various time points and employing physiological and proteomic analyses, the study elucidated the changes in differentially accumulated proteins (DAPs) and the regulatory pathways governing their early responses, thereby providing a foundation for breeding low-temperature-tolerant varieties.

## 2. Results

### 2.1. Photosynthesis and Chlorophyll Response

**Figure 1:**
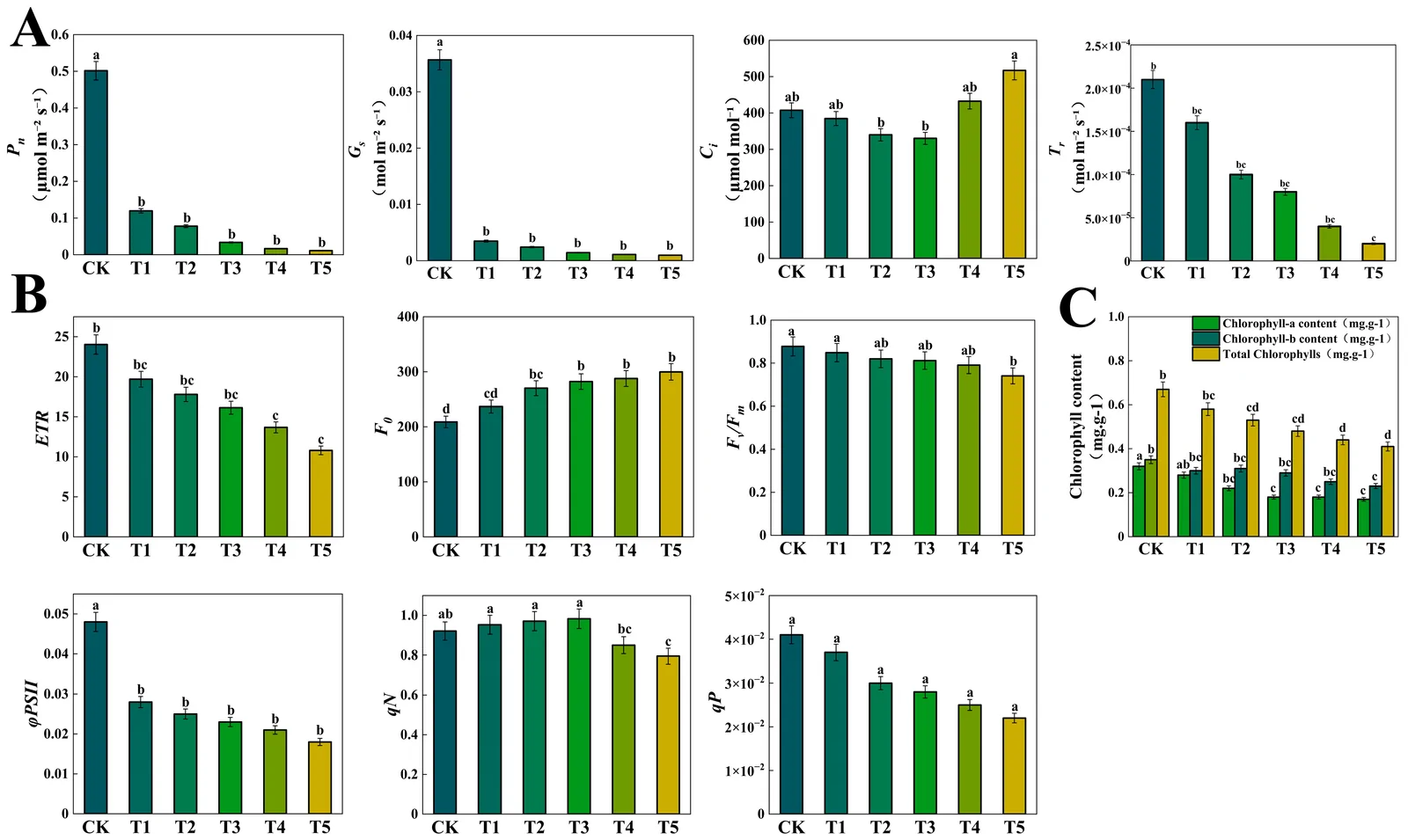
Effects of T1–T5 treatments on photosynthetic parameters, chlorophyll fluorescence parameters, and chlorophyll content. (**A**) Photosynthetic parameters. (**B**) Chlorophyll fluorescence parameters. (**C**) Chlorophyll content. The vertical bar chart shows the standard deviation of three replicates for the same parameter; the different letters above the bars indicate the results of multiple comparisons between treatments T1–T5 (significant differences at *p* < 0.05). Data are presented as mean ± standard deviation. Note: *P_n_*: net photosynthetic rate; *G_s_*: stomatal conductance; *T_r_*: transpiration rate; *C_i_*: intercellular CO_2_ concentration; *ETR*: apparent photosynthetic electron transport rate; *F*_0_: initial fluorescence; *F_v_/F_m_*: maximum photosynthetic efficiency; *ΦPSII:* effective quantum yield of photosystem II; *qN*: non-photosynthetic quenching coefficient; *qP*: photosynthetic quenching coefficient.

Measurements of photosynthetic parameters revealed a pronounced inhibitory effect of low-temperature stress on photosynthetic activity. *P_n_*, *G_s_*, and *T_r_* declined progressively with increasing stress duration, reaching their lowest levels under the T5 treatment, where they corresponded to only 4.11%, 3.14%, and 19.09% of the control (CK), respectively. In contrast, *C_i_* exhibited a biphasic response, with an initial decline followed by a sharp increase, ultimately peaking at T5, which was 7.64% higher than CK (Figure 1A, Table S1). Analysis of chlorophyll fluorescence parameters further confirmed significant impairment of PSII function under low-temperature stress. *F_v_/F_m_*, *ΦPSII*, *qP*, and *ETR* significantly declined to their lowest levels at T5, corresponding to 84.40%, 58.33%, 53.66%, and 44.96% of CK, respectively. *qN* exhibited a pattern of initial increase followed by a decrease, whereas the *F*_0_ increased steadily and reached its maximum value at T5 (1.44-fold higher than CK) (Figure 1B, Table S2). Consistent with these findings, the levels of chlorophyll a, chlorophyll b, and total chlorophyll content decreased significantly with prolonged stress duration, declining to only 53.1%, 65.7% and 61.2% of CK at T5, respectively (Figure 1C, Table S3). Taken together, these results demonstrate that low-temperature stress severely compromises photosynthetic efficiency in oil palm seedlings by impairing PSII activity, disrupting gas exchange dynamics, and markedly reducing chlorophyll biosynthesis.

### 2.2. Membrane Lipid Peroxidation, Permeability Regulation, and Antioxidant Response

**Figure 2:**
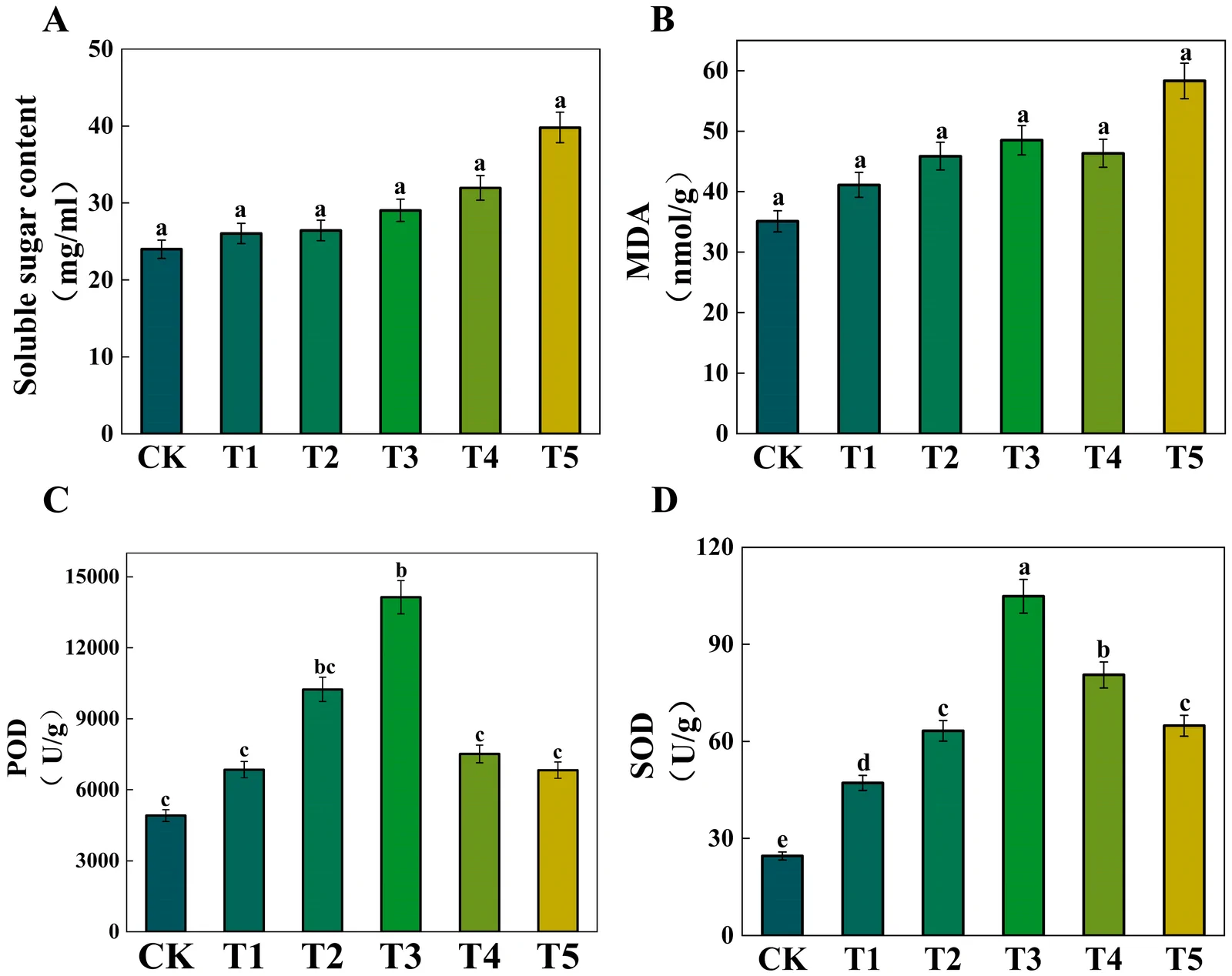
Effects of T1–T5 treatments on plant morphological characteristics, membrane lipid peroxidation, osmoregulation, and antioxidant capacity. (**A**) Soluble sugar content (**B**) MDA content (**C**) POD activity (**D**) SOD activity. The vertical bar chart shows the standard deviation of three replicates for the same parameter; the different letters above the bars indicate the results of multiple comparisons between treatments T1–T5 (significant differences at *p* < 0.05). Data are presented as mean ± standard deviation.

Prolonged exposure to low-temperature stress induced pronounced changes in membrane integrity and antioxidant defenses in oil palm seedlings. An upward trend of soluble sugars and MDA was observed, both of which increased progressively with stress duration (Figure 2A,B). By the end of the stress period (T5 treatment), the levels of soluble sugars and MDA were found to be 1.66-fold higher than those in the control group (CK) (Tables S4 and S5). The activity of antioxidant enzymes exhibited an increase during the initial stress phase (T1–T2 treatment), followed by a gradual decrease in the later phase (T4–T5 treatment) (Figure 2C,D). The peak activities were recorded during the mid-stress period (T3 treatment), reaching 2.88-fold and 4.26-fold of the CK levels, respectively (Tables S6 and S7). However, as the stress intensified (T4–T5 treatments), the activities of both key antioxidant enzymes significantly declined. These results suggest that during the early stages of low-temperature stress, plants respond by accumulating osmotic regulatory substances (soluble sugars) and enhancing the activity of antioxidant enzymes (SOD and POD). Nevertheless, under prolonged or severe stress conditions (T4–T5), the antioxidant defense system becomes impaired, leading to uncontrolled reactive oxygen species accumulation, intensified membrane lipid peroxidation, and aggravated cellular injury.

### 2.3. Correlation of Physiological Indicators

**Figure 3:**
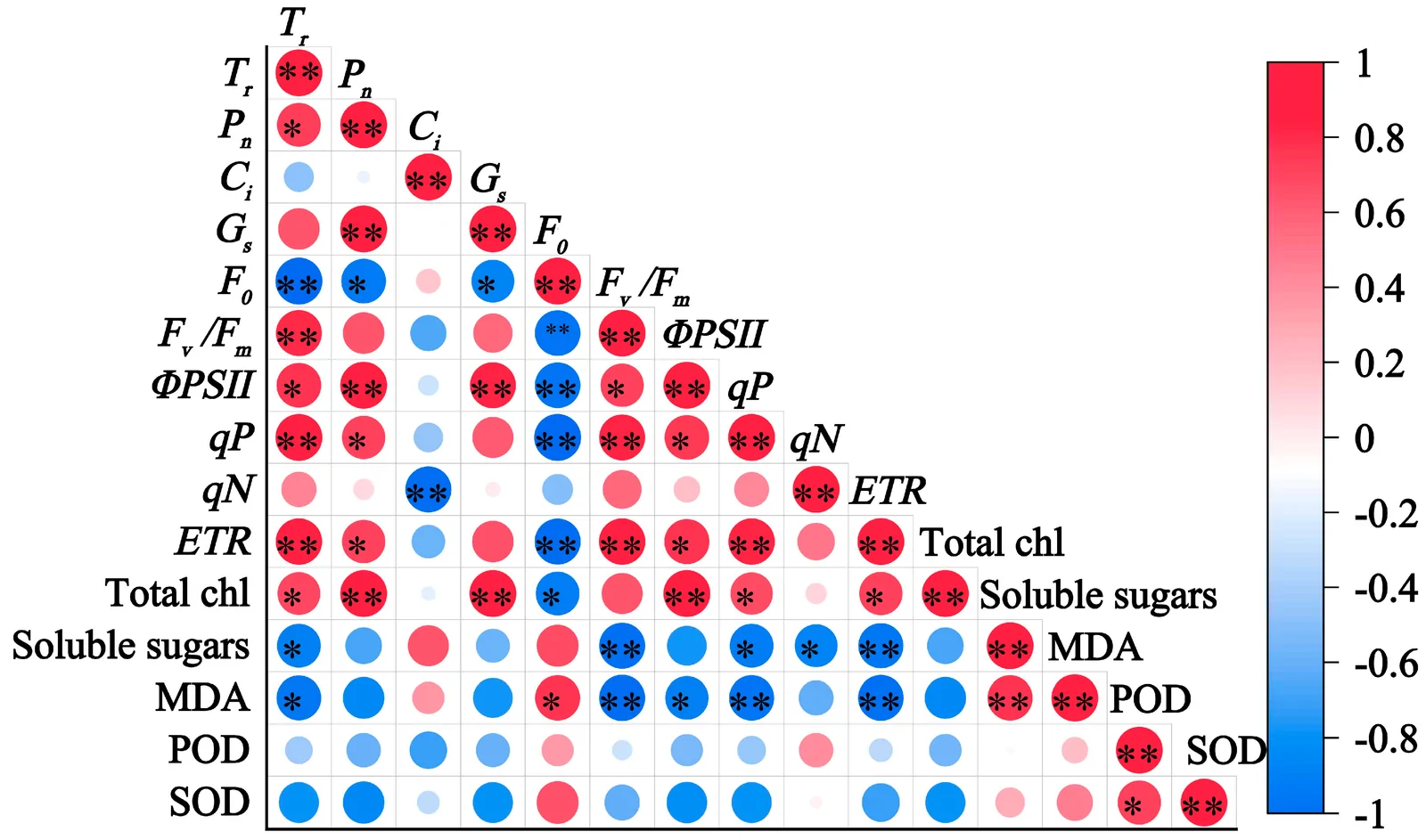
Correlation analysis of physiological indicators. Red indicates high correlation, blue indicates low correlation, and white indicates intermediate values. Note: * Significant correlation at the 0.05 level (two-tailed). ** Significant correlation at the 0.01 level (two-tailed).

A Pearson correlation analysis was conducted to examine the interrelationships among photosynthetic parameters, osmotic adjustment substances, membrane lipid peroxidation products, and antioxidant enzyme activities (Figure 3). The results indicated that PSII was severely inhibited under low-temperature stress, as indicated by a significant increase in *F*_0_, which exhibited strong negative correlations with *F_v_/F_m_*, *ΦPSII*, *qP*, and *P_n_*. In contrast, photosynthetic efficiency was tightly coupled to stomatal conductance and pigment content, with *P_n_*, *G_s_* and total chlorophyll showing highly significant positive correlations. Regarding membrane stability, MDA accumulation was strongly associated with stress-induced damage. MDA displayed significant or extremely significant negative correlations with photosynthetic parameters and chlorophyll content, while showing an extremely significant positive correlation with soluble sugar content. Interestingly, soluble sugars also exhibited significant or highly significant negative correlations with multiple photosynthetic parameters, suggesting that osmotic adjustment occurred in parallel with the decline in photosynthetic activity. In contrast, the antioxidant enzyme system (SOD and POD) appeared to function relatively independently from photosynthetic and osmotic processes. Although SOD and POD activities were positively correlated with each other, neither showed significant associations with other physiological indicators. Taken together, these findings indicate that three physiological changes constitute the core response of oil palm leaves to low-temperature stress: (a) a decline in *ΦPSII*, reflecting PSII inhibition; (b) enhanced accumulation of MDA, indicating exacerbated membrane lipid peroxidation; (c) increased soluble sugar content, contributing to osmotic adjustment. These three tightly linked parameters provide reliable physiological markers of low-temperature injury in oil palm.

### 2.4. Analysis of Protein Abundance Changes

**Figure 4:**
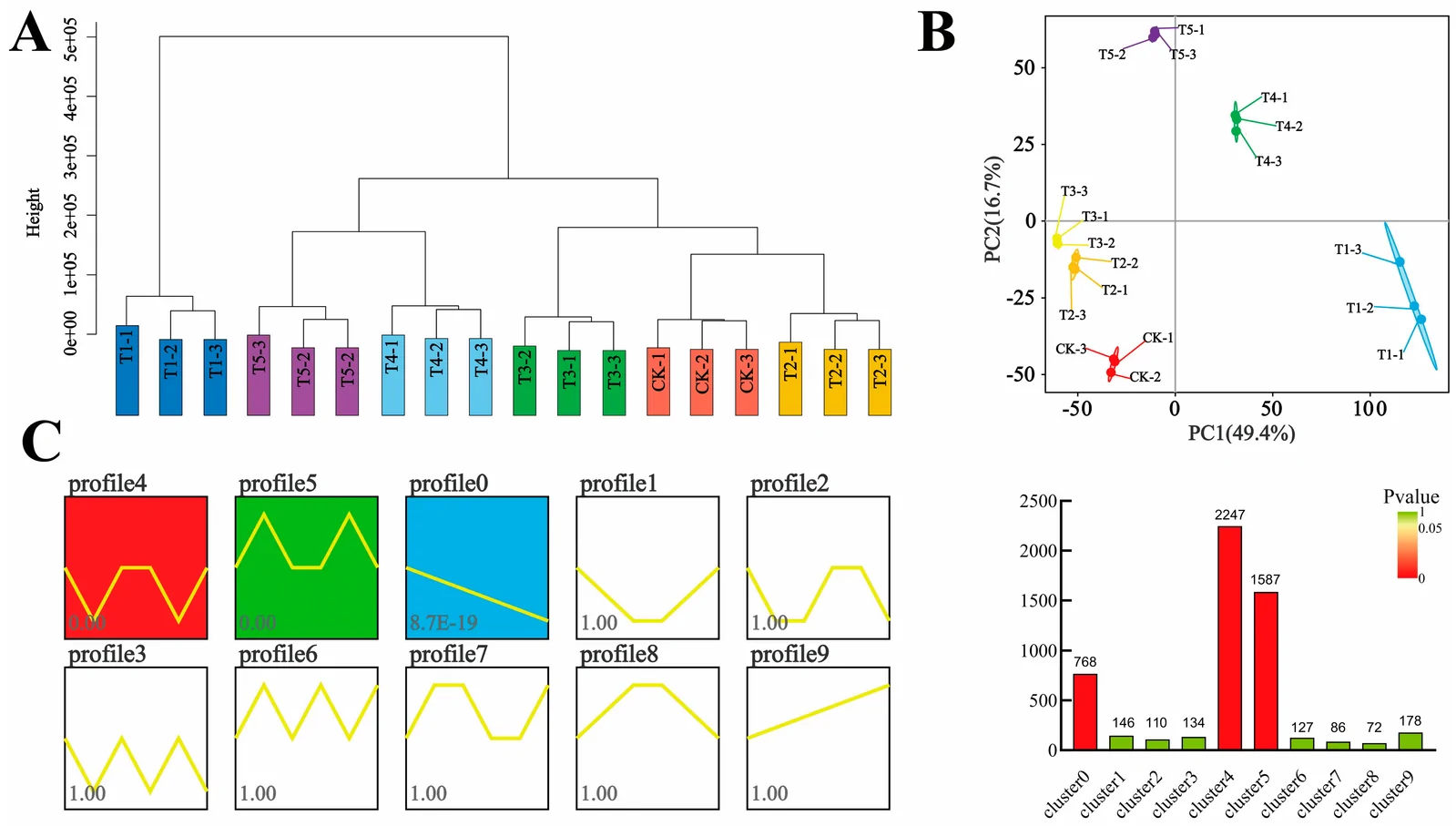
Analysis of changes in protein abundance. (**A**) Cluster analysis of protein abundance. (**B**) PCA analysis of protein changes. (**C**) Trends in protein abundance as determined by STEM, with the vertical axis showing normalized expression levels (log_2_ transformation) and the horizontal axis showing stress time points (CK–T5). The numbers in the bottom left-hand corner represent *p*-values; trends with *p*-values < 0.05 are considered significant. The bar chart on the right shows the protein levels and significance status for each cocoon.

To investigate the molecular mechanisms underlying the oil palm response to low-temperature stress, quantitative proteomics based on TMT labeling was performed. A total of 85,952 peptide segments and 8284 proteins were identified from seedling leaves subjected to low-temperature treatments of varying durations (Table S8). Hierarchical clustering of the protein abundance profiles revealed clear grouping patterns among the treatments (Figure 4A). Long-term stress treatments (T5 and T4 treatment), together with T3, formed a distinct cluster, whereas CK and T2 clustered separately. This indicates that the duration of low-temperature exposure plays a dominant role in shaping protein accumulation patterns, and the high similarity among biological replicates confirms the robustness of the dataset. Principal Component Analysis (PCA) further supported these findings, demonstrating that samples within the same treatment group were highly consistent, while strong separation was observed between groups (Figure 4B). In particular, CK and the T1, T4, and T5 groups displayed the largest divergence, highlighting the pronounced molecular reprogramming triggered during both early and prolonged low-temperature stress.

To explore temporal expression dynamics, Short Time-series Expression Miner (STEM) analysis was conducted (Figure 4C). Proteins were classified into ten expression clusters, of which Clusters 0 (profile0), 4 (profile4), and 5 (profile5) were most enriched. Cluster 0 (profile0) included 768 proteins that were rapidly and continuously downregulated from the onset of stress, suggesting a suppression of basal metabolic activity under cold conditions. In contrast, Cluster 9 (profile9) encompassed 178 proteins that displayed a strong upward trend throughout the stress period, implying their involvement in adaptive responses such as stress signaling, antioxidant defense, and energy reallocation. These contrasting expression modules likely reflect a strategic balance between suppression of growth-related processes and activation of stress-protective pathways. Taken together, these results reveal that low-temperature stress induces highly dynamic and time-dependent proteomic reprogramming in oil palm seedlings, providing a set of candidate proteins that may serve as key regulators of low-temperature stress.

### 2.5. Identification of Differentially Accumulated Proteins and Pathway Enrichment Analysis

**Figure 5:**
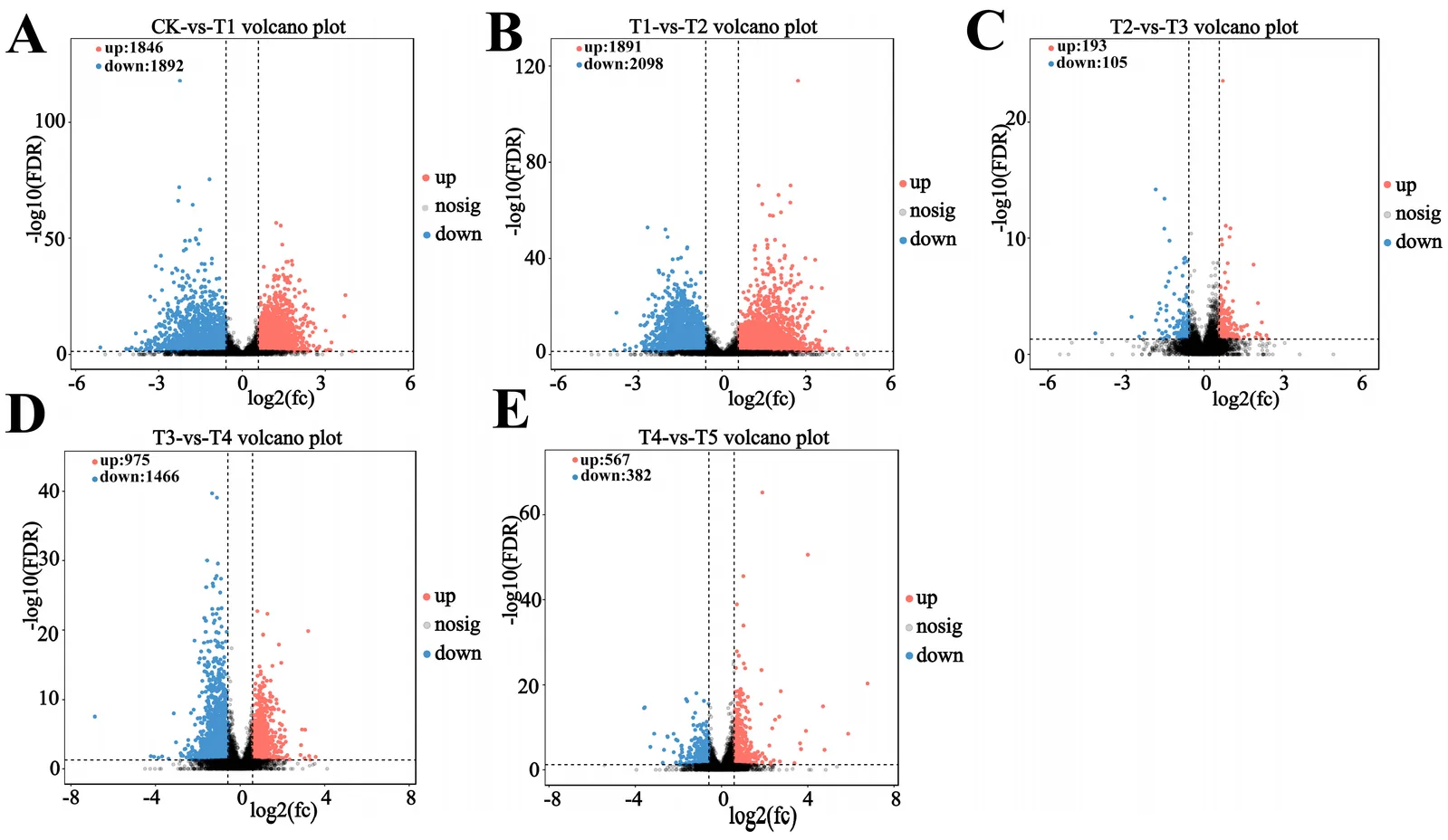
Identification and analysis of DAPs. (**A**–**E**) Volcano plots showing differences in CK_vs._T1, T1_vs._T2, T2_vs._T3, T3_vs._T4, and T4_vs._T5 DAPs. There will be some overlap between the different proteins identified in each period. The x-axis represents the log_2_ fold change (log_2_FC) in protein abundance, whilst the y-axis represents the negative logarithm to base 10 of the FDR value (–log_10_(FDR)), adjusted for significance using the Benjamini–Hochberg method; a higher value indicates a more significant difference after adjustment. In the figure, red dots represent significantly upregulated proteins, blue dots represent significantly downregulated proteins, and grey dots represent proteins with no significant difference. The selection criteria for differentially expressed proteins are that they must simultaneously satisfy FC ≥ 1.5 or ≤0.67 and a corrected FDR < 0.05 (i.e., −log_10_(FDR) > 1.3).

To pinpoint key proteins involved in the oil palm response to low-temperature stress, a differential abundance analysis was conducted across sequential time points (CK_vs._T1, T1_vs._T2, T2_vs._T3, T3_vs._T4, T4_vs._T5) (Figure 5A–E). A total of 5203 DAPs were identified (Table S9). The number of upregulated proteins in the five comparisons was 1846, 1891, 193, 975, and 567, respectively, while downregulated proteins numbered 1892, 2098, 105, 1466, and 382, respectively (Table S10). These results indicate that protein accumulation remodeling is most pronounced during the early stages of stress (CK–T2), reflecting an acute molecular adjustment to cold exposure.

**Figure 6:**
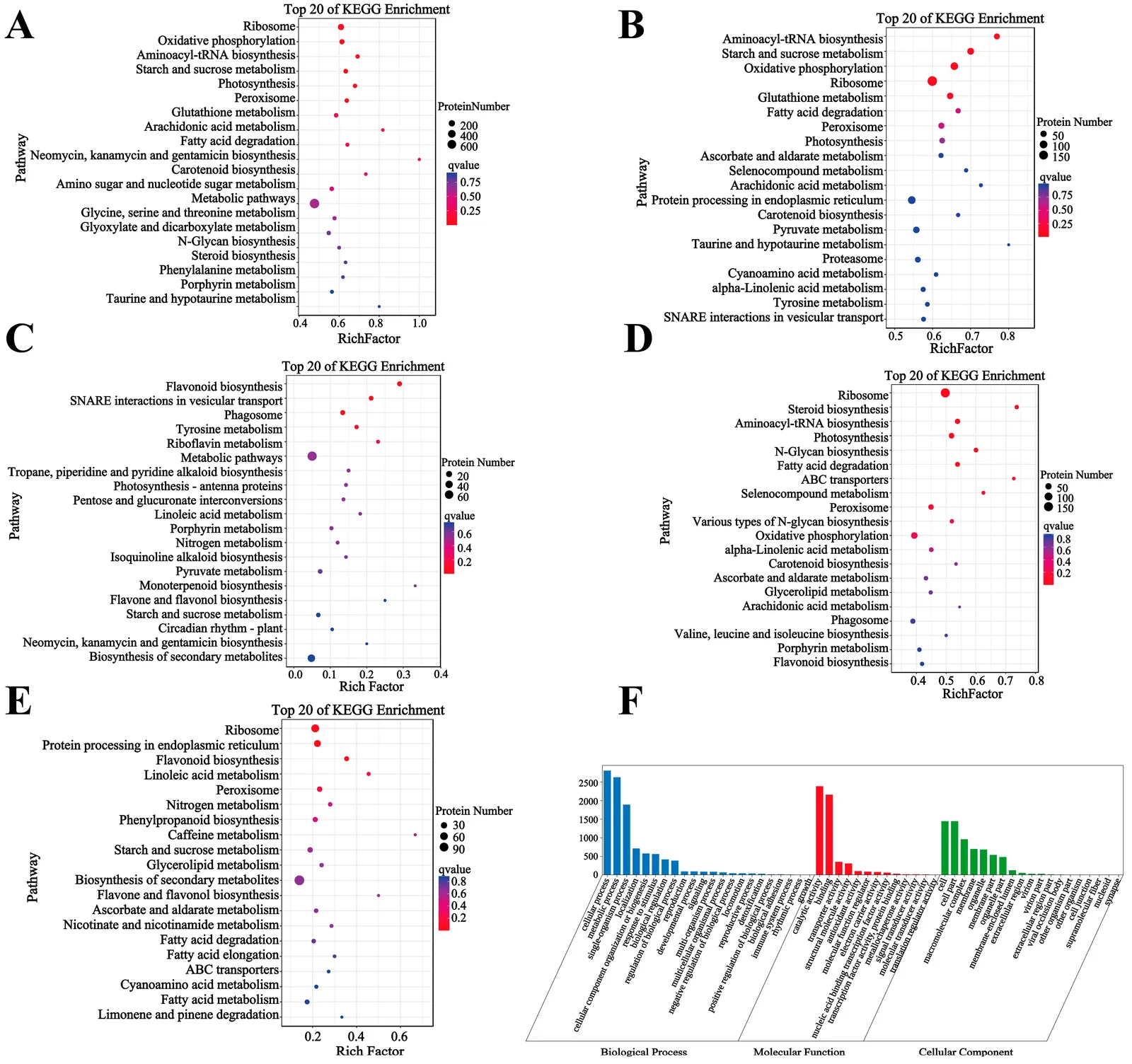
Enrichment analysis of DAPs. (**A**–**E**) KEGG enrichment analysis of the top 20 DAPs for CK_vs._T1, T1_vs._T2, T2_vs._T3, T3_vs._T4, and T4_vs._T5. The vertical axis denotes KEGG enrichment pathways, while the horizontal axis represents enrichment factors. (**F**) Distribution of the number of annotations for all DAPs across GO categories. The vertical axis represents the number of DAPs, and the horizontal axis represents the GO terms. Blue, red, and green bars correspond to biological processes, molecular functions, and cellular components, respectively.

To further investigate the biological processes and metabolic pathways associated with the adaptation of oil palm seedlings to low-temperature stress, 5203 differentially abundant proteins (DAPs) were identified, followed by Gene Ontology (GO) and Kyoto Encyclopedia of Genes and Genomes (KEGG) enrichment analyses. The GO enrichment analysis revealed that the DAPs were significantly enriched in pathways related to membrane lipid repair, ROS metabolic regulation, and immunity. This finding suggests that maintaining cell membrane stability and repairing oxidative damage are crucial for the low-temperature response (Figure 6F, Table S11). KEGG enrichment analysis further revealed that DAPs primarily participate in pathways such as fatty acid degradation, peroxisome function, ribosome biogenesis, and starch and sucrose metabolism (Figure 6A–E). Notably, the pathways of fatty acid degradation and starch and sucrose metabolism are particularly significant, as both are crucial for oil palm low-temperature stress.

Proteomic analysis revealed that oil palm seedlings exhibit a dynamic response to low-temperature stress. During the initial phase of stress (from CK to T2), the most pronounced reorganization of protein abundance occurred. Functional enrichment analysis indicates that these key proteins are primarily involved in pathways related to fatty acid degradation, peroxisome function, ribosome metabolism, and starch and sucrose metabolism. Concurrently, cells maintain membrane stability and repair oxidative damage by activating pathways that govern membrane lipid repair, regulate ROS metabolism, and enhance immunity. Collectively, these findings suggest that the protection of the membrane system, ROS scavenging, and the adjustment of energy metabolism represent core adaptive mechanisms for oil palm seedlings in response to low-temperature stress.

### 2.6. Key Proteins Involved in Fatty Acid Degradation and the Regulation of Starch and Sucrose Metabolism Pathways

**Figure 7:**
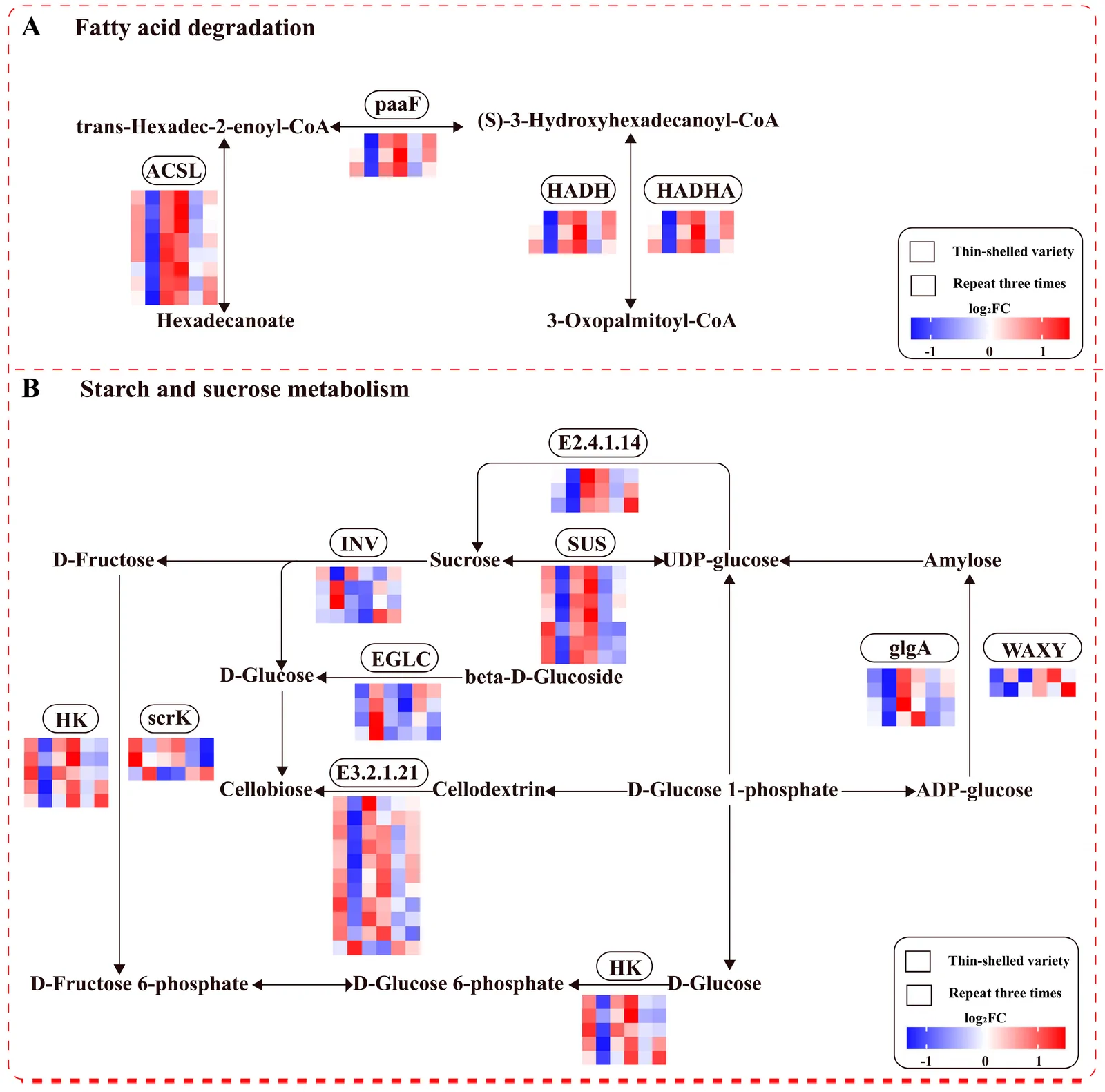
Analysis of metabolic pathways involved in regulation. (**A**) Fatty acid degradation pathways involved in oil palm response to low-temperature stress. (**B**) Starch and sucrose pathways involved in oil palm response to low-temperature stress. Protein heatmap values indicate protein accumulation levels across different samples. Red denotes high accumulation levels; blue denotes low accumulation levels.

Proteomic analysis indicates that low-temperature stress significantly affects fatty acid degradation pathways and starch–sucrose metabolism in oil palm seedlings. KEGG enrichment analysis identified four DAPs involved in the fatty acid degradation pathways: ACSL (long-chain fatty acid-CoA ligase), paaF (enoyl-CoA hydratase), HADH (3-hydroxyacyl-CoA dehydrogenase), and HADHA (enoyl-CoA hydratase/long-chain 3-hydroxyacyl-CoA dehydrogenase) (Figure 7A). These proteins exhibited a notable trend of upregulation and play crucial roles in this metabolic pathway.

In the starch and sucrose metabolic pathways, nine key DAPs were jointly identified: INV (beta-fructofuranosidase), HK (hexokinase), scrK (fructokinase), and EGLC (glucan endo-1,3-beta-D-glucosidase), among others. Notably, INV (beta-fructofuranosidase), EGLC (glucan endo-1,3-β-D-glucosidase), WAXY (granule-bound starch synthase), E3.2.1.21 (beta-glucosidase), and E2.4.1.14 (sucrose-phosphate synthase) exhibited significant upregulation, whereas glgA (starch synthase) and SUS (sucrose synthase) demonstrated significant downregulation. HK (hexokinase) and scrK (fructokinase) displayed an initial increase followed by a decrease (Figure 7B). The complex expression patterns of these differentially accumulated proteins reveal the dynamic network of this regulatory pathway in response to low-temperature stress.

## 3. Discussion

### 3.1. Physiological Adaptation Mechanisms of Oil Palm Seedlings Under Low-Temperature Stress

This thorough examination reveals that the main limitation affecting the physiological adaptation of oil palm seedlings to low-temperature stress is the considerable reduction in photosynthesis. Low-temperature conditions lead to marked declines in *P_n_*, *G_s_*, and *T_r_*. In the initial stages, there is a decrease in *C_i_*, indicating that stomatal limitations are primarily responsible for the reduction in photosynthesis. Conversely, in the later stages, *C_i_* increases even as *P_n_* sharply drops, implying that non-stomatal elements—especially the structural and functional damage to photosystem II (PSII)—predominate in the inhibition of photosynthesis [13,26]. Analysis of chlorophyll fluorescence further supports the notion of PSII reaction center inactivation, disruption within the electron transport chain, and decreased photochemical efficiency, ultimately resulting in diminished light energy conversion [27,28]. The decline in photosynthetic performance was also associated with significant changes in the levels of chlorophyll a, b, and total chlorophyll. Correlation assessments indicated a strong positive relationship among *P_n_*, *G_s_*, and total chlorophyll, affirming that chlorophyll degradation plays a pivotal role in photosynthetic inhibition, thus establishing chlorophyll as a dependable indicator of both stress severity and photosynthetic performance [29,30].

Apart from the limitations imposed by photosynthesis, key elements such as damage from membrane lipid peroxidation and imbalances in osmotic regulation play vital roles in how organisms respond to low-temperature conditions. The ongoing buildup of MDA serves as an indicator of advancing membrane lipid peroxidation, exhibiting strong negative correlations with both photosynthetic metrics and chlorophyll levels. This highlights the crucial involvement of ROS-induced oxidative harm in precipitating overall physiological deterioration [31,32]. Although soluble sugars are often associated with osmotic regulation, their increasing levels were found to correlate significantly with MDA and inversely with photosynthetic characteristics. This implies that during periods of low temperature, the accumulation of sugars might be more indicative of damage-related byproducts rather than an effective defense strategy [33]. Additionally, the enzyme activities of SOD and POD demonstrated no meaningful correlation with other physiological metrics, suggesting that the antioxidant system does not possess adequate capacity to alleviate prolonged ROS stress, which allows for the buildup of oxidative damage [34,35,36]. Moreover, the significant positive relationship between *P_n_* and total chlorophyll content further reinforces the notion that the rapid malfunction of the photosynthetic system serves as a key catalyst for excessive ROS generation, which in turn triggers a series of oxidative damage processes [37,38,39].

In conclusion, the physiological vulnerability of oil palm seedlings to low-temperature stress can be attributed to three interconnected limitations: (i) rapid breakdown of the photosynthetic machinery leading to photosynthetic inhibition, (ii) progressive membrane lipid peroxidation and insufficient ROS detoxification capacity resulting in a cascade of oxidative damage, and (iii) limited efficacy of osmotic regulation. Together, these factors provide a mechanistic framework for understanding the intrinsic cold sensitivity of oil palm seedlings and emphasize potential targets for breeding and biotechnological interventions to enhance low-temperature stress.

### 3.2. The Pivotal Role of Fatty Acid Degradation Pathways in Enhancing Oil Palm Low-Temperature Stress

Low-temperature stress is a significant environmental factor that limits both the cultivation range and yield of oil palms. The primary damage caused by this stress arises from the disruption of the cell membrane system and the subsequent metabolic disorders [40,41]. This study, grounded in proteomics analysis, reveals that the fatty acid degradation pathway, also known as the fatty acid β-oxidation pathway, is significantly activated during the low-temperature response process. This pathway may represent a key regulatory mechanism through which oil palm responds to low-temperature stress. It is hypothesized that this pathway enhances the plant’s low-temperature tolerance via a combination of mechanisms: rapidly breaking down lipids to provide energy, regulating cell membrane fluidity to maintain integrity, and synergistically activating antioxidant systems and hormonal signaling networks. The activation of this pathway may serve as a crucial foundation for oil palm’s response to low-temperature stress, functioning through multiple mechanisms, including providing emergency energy to cells, maintaining the stability of membrane systems, and regulating redox balance [42,43,44].

Under low-temperature stress, proteins such as ACSL, paaF, HADH, and HADHA exhibited significant accumulation. The synergistic upregulation of these proteins indicates that oil palms may sustain membrane lipid homeostasis and energy balance by promoting fatty acid degradation metabolism, thereby improving low-temperature stress. It is hypothesized that the fatty acid β-oxidation pathway serves not only as a vital energy supply route but also that its products may function as signaling molecules or precursors for membrane lipid remodeling. These molecules are involved in cold signal transduction and cell membrane repair, playing a significant role in the early response to low-temperature stress in plants [45,46,47]. According to previous research, it has been demonstrated that ACSL, as the key enzyme for fatty acid activation, catalyzes the binding of long-chain fatty acids with coenzyme A to form acyl-CoA, thereby providing substrates for subsequent β-oxidation. Its upregulation suggests that oil palms may enhance fatty acid activation under low temperatures, promoting their entry into degradation pathways. This process facilitates rapid acetyl-CoA production and its integration into the TCA cycle (tricarboxylic acid cycle), thereby sustaining the energy supply [48]. Other key enzymes within this pathway perform essential functions. Therefore, we propose that the upregulation of these enzymes not only enhances energy metabolic efficiency but may also influence the composition and fluidity of membrane lipids by regulating the accumulation of lipid metabolic intermediates. This regulation, in turn, strengthens the stability of the cell membrane under low-temperature conditions, thereby ensuring the normal growth of oil palm.

The molecular-level alterations observed in this study exhibit a correlation with the physiological indicators assessed. Low-temperature stress resulted in a reduction in chlorophyll content in oil palm leaves, accompanied by a sustained decline in photosynthetic parameters, indicating a severe impairment of photosynthetic capacity. In this context, the activation of the fatty acid β-oxidation pathway can be regarded as a crucial metabolic compensatory strategy, providing cells with the energy necessary to maintain basal metabolism and repair damage. Simultaneously, during the mid-stress phase, antioxidant enzyme activity peaked, indicating a strong induction of the antioxidant system. This, along with the oxidative signals potentially generated by fatty acid oxidation, collectively constituted a defense response [49,50]. However, as stress intensified, despite the sustained high accumulation of enzymes involved in the fatty acid β-oxidation pathway, MDA levels continued to accumulate, while antioxidant enzyme activity declined. This suggests that the damage caused by membrane lipid peroxidation progressively exceeded the cell’s capacity for repair and clearance [49,50]. This indicates that while the fatty acid β-oxidation pathway contributes to alleviating low-temperature stress, the full realization of its protective effects likely depends on synergistic interactions with multiple pathways, including the antioxidant system and membrane repair mechanisms.

In summary, the fatty acid β-oxidation pathway may represent a crucial regulatory step in the response of oil palm to low-temperature stress. Further research is necessary to elucidate the roles of ACSL, paaF, HADH, and HADHA, thereby clarifying their potential as targets for breeding low-temperature tolerance.

### 3.3. The Pivotal Role of Starch and Sucrose Metabolic Pathways in Enhancing Oil Palm Low-Temperature Stress

Low-temperature stress significantly disrupts the balance of carbon metabolism in plants. Among these pathways, starch and sucrose metabolism serve as core mechanisms for energy supply and osmotic regulation, playing a pivotal role in plants’ responses to abiotic stresses, including low temperatures, drought, and salinity [51,52,53]. This study identified nine dynamic alterations in the DAPs within this pathway. These findings suggest that, when exposed to low-temperature stress, oil palm seedlings may reprogram their carbon allocation strategy through the precise regulation of multiple key enzymes involved in starch and sucrose metabolism. Such adaptations are crucial for maintaining cellular energy homeostasis and osmotic equilibrium, thereby enhancing tolerance to low-temperature stress [54,55,56].

From the perspective of coordinated changes in physiological indicators and protein accumulation, it can be inferred that the adjustment of starch and sucrose metabolic pathways is closely associated with the decline in photosynthesis and the processes of membrane lipid peroxidation in oil palms under low-temperature conditions [18,57]. This study demonstrates that as the duration of stress prolongs, the photosynthetic systems become impaired, leading to an increase in soluble sugar content. This phenomenon indicates that when photosynthetic mechanisms are compromised, oil palms mobilize intracellular reserve carbon sources by enhancing starch degradation and sucrose synthesis pathways to sustain essential metabolic demands and osmotic equilibrium [58,59]. The expression changes in key enzymes support this adaptive mechanism: the upregulation of INV may enhance stress resistance by participating in cell wall biosynthesis [60]. ScrK, a key enzyme in fructose phosphorylation, exhibits an expression pattern that initially increases and then decreases, potentially reflecting a diminished regulatory capacity during the later stages of stress [61]. The upregulation of EGLC may promote starch hydrolysis and soluble sugar accumulation, thereby enhancing low-temperature stress [35]. Additionally, the upregulation of E3.2.1.21 may contribute to osmotic regulation and reactive oxygen species stabilization, helping mitigate the effects of abiotic stress [62]. Furthermore, the increase in E2.4.1.14 during short-term stress may support sucrose synthesis and energy supply [63]. The key sucrose metabolism enzyme, SUS, is involved in both sucrose degradation and synthesis, playing a crucial role in starch accumulation, stress resistance, and plant growth [61]. Its downregulated expression may indicate that low-temperature stress disrupts the normal growth processes of oil palms. Furthermore, studies on *Ulmus pumila* suggest that WAXY and glgA may enhance stress adaptation by promoting starch accumulation, regulating energy metabolism, and boosting photosynthesis [64]. Despite its significance, HK has received limited attention in previous studies on low-temperature stress. Overall, although these enzymes have been investigated in the stress responses of other plants, their specific functions in the low-temperature stress of oil palms require further validation.

In summary, the oil palm adapts to low-temperature stress by dynamically regulating the expression of several key enzymes within the starch and sucrose metabolic pathways. It is hypothesized that these changes may play a role in mobilizing stored carbon sources, maintaining energy supply, and ensuring osmotic balance, thereby mitigating photosynthetic damage and enhancing low-temperature tolerance. However, the specific functions of the relevant DAPs in the oil palm require further validation.

## 4. Materials and Methods

### 4.1. Experimental Materials and Processing

The experimental materials consisted of one-year-old potted seedlings of thin-shelled oil palm, provided by the Wenchang National Tropical Palm Germplasm Repository in Hainan Province, China. Seedlings were selected based on the following criteria: vigorous growth, uniform size and morphology, absence of visible diseases or pests, and similar developmental stages (≥six fully expanded true leaves). Before stress treatment, all seedlings were acclimatized under controlled environmental conditions in an LEDR-1000 intelligent artificial climate chamber (Ningbo Yanghui Instrument Co., Ltd., Ningbo, Zhejiang, China). The chamber was equipped with a full-spectrum white LED light source (spectral range, 400–700 nm; red-to-blue light ratio, approximately 3:1) and programmed with a 16 h light/8 h dark photoperiod. Light intensity was monitored in real time at the seedling canopy height using an LI-190R quantum sensor (LI-COR, Lincoln, NE, USA) and maintained at 300 ± 10 µmol m^−2^ s^−1^. The temperature was maintained at 25 ± 1 °C, and relative humidity was maintained at 60–70%.

Low-temperature stress was subsequently imposed in the same LEDR-1000 intelligent climate chamber, with all environmental parameters maintained at the same levels as during acclimatization except for temperature. The chamber temperature was set to 8 °C, and five stress-treatment time points were established: 0.5 h (T1), 1 h (T2), 2 h (T3), 4 h (T4), and 8 h (T5), in addition to a control group (CK) maintained at 25 ± 1 °C. Light intensity was continuously monitored and maintained at a constant level throughout the stress treatment using the LI-190R quantum sensor to minimize potential physiological effects caused by fluctuations in light intensity. Thus, six experimental groups (CK and T1–T5) were established, with three biological replicates per group, resulting in a total of 18 seedlings. Each seedling represented one biological replicate. Multiple functional leaves were collected from each seedling from the same position and at a comparable developmental stage and pooled in equal proportions to generate the sample for each biological replicate. The leaf samples were immediately flash-frozen in liquid nitrogen and stored at −80 °C until subsequent proteomic and biochemical analyses.

### 4.2. Measurement of Photosynthetic Parameters

After applying low-temperature treatments, we assessed the impact of stress on leaf photosynthetic performance by measuring photosynthetic and chlorophyll fluorescence parameters. These measurements were conducted between 6:00 and 14:00 under natural light in a plant photosynthesis growth chamber. For consistent observations, three plants of comparable size and growth were chosen from each treatment group. The second fully expanded mature leaf situated just below the terminal leaf was analyzed for determining photosynthetic parameters. The evaluation of gas exchange parameters was conducted using a portable photosynthesis system (LI-6800, LI-COR Biosciences, Lincoln, NE, USA). Parameters measured included the *P_n_*, *G_s_*, *T_r_*, and *C_i_*. Additionally, light-adapted chlorophyll fluorescence parameters were documented using the same equipment, focusing on the apparent photosynthetic electron transport rate (*ETR*), *ΦPSII*, *qP*, and *qN*. For dark-adapted fluorescence measurements, the third mature leaf beneath the terminal leaf was dark-adapted for 30 min using shading clips. We subsequently assessed the *F*_0_ and *F_v_/F_m_* to evaluate the photosynthetic apparatus’s integrity under low-temperature stress.

### 4.3. Measurement of Physiological Indicators

To evaluate the physiological responses of oil palm seedlings under low-temperature stress, several key physiological parameters were determined. Chlorophyll content, MDA content, soluble sugar content., and the activities of antioxidant enzymes, including SOD and POD, were quantified using commercially available assay kits (Solarbio Science & Technology Co., Ltd., Beijing, China).

### 4.4. Protein Extraction and Analysis

Proteins were obtained from samples of oil palm seedlings through the trichloroacetic acid (TCA)/acetone precipitation technique [65]. The concentration of proteins was assessed using the Bradford Protein Assay Kit (Solarbio, Beijing, China) [66]. For digestion, 200 μg of protein from each sample underwent processing via the filter-aided sample preparation (FASP) method. Peptides were tagged with 10-plex tandem mass tag (TMT) reagents (Thermo Fisher Scientific, Art. No. 90111, Waltham, MA, USA) according to the directions provided by the manufacturer, utilizing 100 μg of peptides from each sample. For the reduction and alkylation steps, protein lysates were combined with 10 mM tris(2-carboxyethyl)phosphine (TCEP) and kept at 37 °C for 60 min, followed by a treatment with 40 mM iodoacetamide at room temperature in the dark for 40 min. Next, six volumes of cold acetone were added to the mixture, causing protein precipitation at −20 °C for 4 h, followed by centrifugation at 10,000× *g* for 20 min at 4 °C. The resultant pellets were resuspended in 100 μL of 50 mM triethylammonium bicarbonate (TEAB) buffer, allowed to digest overnight at 37 °C using trypsin at a 1:50 (enzyme:protein) ratio, and labeled with TMT reagents for 2 h at room temperature. The labeling reaction was stopped with hydroxylamine for 15 min. This experiment comprised a total of 18 samples, which included 6 treatment groups and 3 biological replicates each. Since each batch of 10-plex TMT reagent can label a maximum of 10 samples, the labeling was conducted in two batches. An internal control channel was mixed into each batch to facilitate inter-batch signal correction. After labeling was completed, all samples were pooled, desalted, and vacuum-dried.

Peptides labeled with TMT were separated into 10 fractions using reverse-phase high-performance liquid chromatography (Agilent 1260 Infinity II, Agilent Technologies, Santa Clara, CA, USA). These fractions were then vacuum-dried and analyzed via nano-liquid chromatography tandem mass spectrometry (nano-LC-MS/MS) with an EASY-nLC system coupled to a Q Exactive Plus mass spectrometer (Thermo Fisher Scientific). The mass spectrometer operated in positive ion mode using a data-dependent acquisition strategy (top 10). Survey scans were conducted over a mass range of 350–1800 *m*/*z* with a resolution of 70,000 (AGC target, 3 × 10^6^; maximum IT, 50 ms). MS2 spectra were generated using higher-energy collisional dissociation (HCD) at a resolution of 17,500 (AGC target, 2 × 10^5^; maximum IT, 45 ms; isolation window, 2 *m*/*z*; normalized collision energy, 30 eV). A dynamic exclusion time of 30 s was implemented, and ions with charge states ranging from 2 to 6 and intensities of at least 2 × 10³ were selected for fragmentation.

The MS/MS spectra were analyzed utilizing Proteome Discoverer 2.2 (Thermo Fisher Scientific) along with the MASCOT search engine (Matrix Science, London, UK) against the protein database of oil palm (https://genhua.tll.org.sg/, accessed on 13 April 2026, CNGBdb: CNA0047477). Proteins were identified by maintaining a false discovery rate (FDR) of less than 1%, requiring a minimum of two unique peptides and a sequence coverage of at least 5%. DAPs were identified based on a fold change of FC ≥ 1.5 or ≤0.67 and a *p*-value of less than 0.05.

### 4.5. Data Processing

All physiological parameters are expressed as mean ± standard deviation (SD). Prior to data analysis, the Shapiro–Wilk test was employed to assess normality, while Levene’s test was utilized to evaluate the homogeneity of variances. When the conditions were satisfied, one-way analysis of variance (ANOVA) was conducted, supplemented by Tukey’s HSD test for post hoc multiple comparisons. In cases where homogeneity of variances was not achieved, the Games–Howell test was applied. All statistical analyses were performed using SPSS Statistics v27.0 (IBM Corp., Armonk, NY, USA) and Microsoft Excel 2021 (Microsoft Corp., Redmond, WA, USA). For the identification of differentially abundant proteins (DAPs), a two-tailed t-test was executed on the quantitative values for each protein, and the Benjamini–Hochberg method was employed to control the false discovery rate (FDR), with thresholds set at q < 0.05 and fold change (FC) ≥ 1.5 or ≤0.67 for statistical significance. Data visualization was conducted using Origin Pro 2024 (Origin Lab, Northampton, MA, USA), GraphPad Prism v10 (GraphPad Software, San Diego, CA, USA), and TBtools v2.05. Venn diagrams and clustering heatmaps were generated using the Kidio online cloud platform (https://www.example.comls/, accessed on 13 April 2026).

## 5. Conclusions

The physiological and molecular response mechanisms of plants to low-temperature stress are intricate. This study systematically elucidates the physiological and molecular regulatory mechanisms that underlie the responses of oil palm seedlings to low-temperature stress through integrated physiological and proteomic analyses. Physiological analyses indicate that low-temperature stress significantly inhibits seedling photosynthesis, resulting in reduced chlorophyll content. Concurrently, membrane lipid peroxidation intensifies, osmotic regulatory substances accumulate, and antioxidant enzyme activities exhibit fluctuating changes. Proteomic differential analysis identifies 5203 DAPs, which are predominantly enriched in pathways related to fatty acid degradation and the regulation of starch and sucrose metabolism. Analysis of key metabolic pathways revealed significantly upregulated DAPs, including ACSL, paaF, HADH, and HADHA, in the fatty acid degradation pathway. It is hypothesized that these proteins play a crucial role in maintaining cellular homeostasis by enhancing metabolic efficiency. In the starch and sucrose metabolism pathways, proteins such as INV, EGLC, and WAXY exhibited DAPs. It is proposed that these proteins may respond to low-temperature stress by mobilizing reserve carbon sources, maintaining energy supply, and regulating osmotic balance. These findings indicate that the fatty acid degradation and starch–sucrose metabolism pathways are significant in the context of oil palm low-temperature stress, although the specific functions of the associated enzymes require further validation. In subsequent experiments, we will establish a comprehensive parallel control group at room temperature, alongside experiments conducted at varying temperatures, to validate the key upregulated DAPs. The findings of this study enhance our understanding of the early responses of plants to low temperatures and provide a theoretical foundation for future molecular breeding and variety selection aimed at improving low-temperature tolerance.

## Data Availability

The original contributions presented in this study are included in the article/Supplementary Material. Further inquiries can be directed to the corresponding authors.

## Supplementary Materials

The following supporting information can be downloaded at: https://www.mdpi.com/article/10.3390/ijms27167418/s1.
