# Supplementary material for: An Analysis of the Early Responses to Low-Temperature Stress in Oil Palm Using Integrative Physiology and Proteomics
Source: Int J Mol Sci. 2026 Aug 19;27(16):7418. doi: 10.3390/ijms27167418 (PMC13513835; doi:10.3390/ijms27167418)
Supplement: Supplementary file 1 [file ijms-27-07418-s001.zip › ijms-4493913-supplementary Tables S1-S7.pdf]

Supplementary File

Table S1 Photosynthetic parameters

Sample	Tr（mol m^{-2} s^{-1}）	Pn（μmol m^{-2} s^{-1}）	Ci（μmol mol^{-1}）	Gs（mol m^{-2} s^{-1}）
**CK1**	0.00021	0.46204	421.09216	0.03094
**CK2**	0.00011	0.38990	437.13221	0.05590
**CK3**	0.00030	0.65278	364.07883	0.02015
**T1-1**	0.00029	0.06216	381.34492	0.00151
**T1-2**	0.00008	0.16393	364.11222	0.00396
**T1-3**	0.00012	0.13267	408.63790	0.00501
**T2-1**	0.00010	0.05127	314.88121	0.00459
**T2-2**	0.00016	0.04998	335.20114	0.00150
**T2-3**	0.00006	0.13260	369.95188	0.00117
**T3-1**	0.00012	0.01536	389.93306	0.00119
**T3-2**	0.00005	0.04851	346.54291	0.00030
**T3-3**	0.00006	0.03703	253.94457	0.00283
**T4-1**	0.00005	0.01494	470.45104	0.00037
**T4-2**	0.00002	0.03229	304.55981	0.00099

**T4-3**	0.00004	0.00289	522.61210	0.00198
**T5-1**	0.00001	0.00580	462.38162	0.00036
**T5-2**	0.00004	0.01862	517.67767	0.00215
**T5-3**	0.00001	0.00907	571.11291	0.00041

Table S2 Chlorophyll fluorescence parameters

Sample	F0	Fv /Fm	φPSII	qP	qN	ETR
**CK1**	218.940	0.993	0.046	0.036	0.853	29.494
**CK2**	206.819	0.781	0.055	0.058	0.961	24.033
**CK3**	200.837	0.860	0.041	0.029	0.948	18.572
**T1-1**	232.235	0.821	0.028	0.048	0.936	21.129
**T1-2**	262.850	0.894	0.035	0.027	0.970	13.867
**T1-3**	215.464	0.833	0.020	0.035	0.951	24.095
**T2-1**	281.946	0.818	0.033	0.037	0.993	17.221
**T2-2**	261.057	0.813	0.022	0.017	0.979	16.783
**T2-3**	267.588	0.828	0.019	0.035	0.940	19.410
**T3-1**	282.747	0.815	0.019	0.015	0.996	14.931
**T3-2**	270.700	0.789	0.025	0.036	0.987	15.605

**T3-3**	293.104	0.831	0.024	0.032	0.966	17.873
**T4-1**	296.711	0.784	0.024	0.028	0.858	13.497
**T4-2**	275.455	0.793	0.020	0.030	0.859	15.509
**T4-3**	291.078	0.796	0.020	0.017	0.834	12.044
**T5-1**	307.072	0.709	0.021	0.020	0.752	11.956
**T5-2**	286.161	0.718	0.018	0.032	0.845	9.747
**T5-3**	306.099	0.797	0.017	0.012	0.788	10.714

Table S3 Chlorophyll content

Sample	weight/g	Wavelength (645 nm)	Wavelength (663 nm)	Chlorophyll a content(mg/g)	Chlorophyll a content(mg/g)	Total chlorophyll content(mg/g)
**CK1**	0.101	0.124	0.195	0.35	0.32	0.67
**CK2**	0.103	0.148	0.179	0.30	0.41	0.72
**CK3**	0.104	0.125	0.173	0.30	0.33	0.63
**T1-1**	0.104	0.104	0.138	0.24	0.28	0.51
**T1-2**	0.102	0.138	0.184	0.32	0.38	0.70
**T1-3**	0.102	0.097	0.159	0.29	0.24	0.53
**T2-1**	0.104	0.115	0.126	0.21	0.33	0.53
**T2-2**	0.101	0.106	0.129	0.22	0.30	0.52
**T2-3**	0.1	0.109	0.131	0.23	0.31	0.54

**T3-1**	0.1	0.099	0.101	0.17	0.30	0.47
**T3-2**	0.1	0.097	0.111	0.19	0.28	0.48
**T3-3**	0.101	0.102	0.109	0.18	0.30	0.48
**T4-1**	0.102	0.095	0.106	0.18	0.27	0.45
**T4-2**	0.1	0.088	0.1	0.17	0.26	0.43
**T4-3**	0.104	0.085	0.116	0.20	0.23	0.42
**T5-1**	0.1	0.093	0.105	0.18	0.27	0.45
**T5-2**	0.101	0.082	0.1	0.17	0.23	0.41
**T5-3**	0.102	0.071	0.092	0.16	0.20	0.36

Table S4 Soluble sugar content

Sample	weight/g	Measurement（620nm）	ΔA	Sample concentration	content（mg/ml）
**CK1**	0.103	0.016	-0.052	0.03	3.31
**CK2**	0.100	0.108	0.040	0.43	43.08
**CK3**	0.101	0.068	0.000	0.26	25.57
**T1-1**	0.101	0.055	-0.013	0.20	20.02
**T1-2**	0.103	0.092	0.024	0.36	35.13

**T1-3**	0.103	0.063	-0.005	0.24	22.98
**T2-1**	0.102	0.110	0.042	0.44	43.08
**T2-2**	0.101	0.040	-0.028	0.14	13.62
**T2-3**	0.101	0.061	-0.007	0.23	22.59
**T3-1**	0.105	0.053	-0.015	0.19	18.44
**T3-2**	0.103	0.044	-0.024	0.15	15.03
**T3-3**	0.102	0.135	0.067	0.55	53.65
**T4-1**	0.101	0.093	0.025	0.37	36.25
**T4-2**	0.104	0.071	0.003	0.27	26.08
**T4-3**	0.104	0.089	0.021	0.35	33.54
**T5-1**	0.105	0.094	0.026	0.37	35.28
**T5-2**	0.100	0.122	0.054	0.49	49.12
**T5-3**	0.102	0.091	0.023	0.36	35.05

Table S5 MDA content

**Sample**	**weight（g）**	**Measurement（532nm）**	**Measurement（600nm）**	**ΔA532nm**	**ΔA600nm**	**ΔA**	**content（nmol/g）**
**CK1**	0.101	0.173	0.067	0.129	0.026	0.103	32.90

**CK2**	0.101	0.180	0.059	0.136	0.018	0.118	37.69
**CK3**	0.104	0.194	0.079	0.15	0.038	0.112	34.74
**T1-1**	0.104	0.196	0.102	0.152	0.061	0.091	28.23
**T1-2**	0.103	0.269	0.146	0.225	0.105	0.12	37.58
**T1-3**	0.102	0.292	0.107	0.248	0.066	0.182	57.56
**T1-1**	0.101	0.284	0.082	0.24	0.041	0.199	63.56
**T1-2**	0.105	0.159	0.054	0.115	0.013	0.102	31.34
**T1-3**	0.105	0.248	0.106	0.204	0.065	0.139	42.70
**T2-1**	0.105	0.215	0.103	0.171	0.062	0.109	33.49
**T2-2**	0.1	0.285	0.091	0.241	0.05	0.191	61.61
**T2-3**	0.103	0.257	0.093	0.213	0.052	0.161	50.42
**T4-1**	0.101	0.277	0.154	0.233	0.113	0.12	38.33
**T4-2**	0.101	0.276	0.131	0.232	0.09	0.142	45.35
**T4-3**	0.102	0.285	0.107	0.241	0.066	0.175	55.34
**T8-1**	0.105	0.295	0.115	0.251	0.074	0.177	54.38
**T8-2**	0.101	0.343	0.115	0.299	0.074	0.225	71.86
**T8-3**	0.102	0.305	0.148	0.261	0.107	0.154	48.70

Table S6 POD activity

**Sample**	**weight（g）**	**Measurement [470nm（30S)]**	**Measurement [470nm(90S)]**	**ΔA**	**Activity(U/g)**
**CK1**	0.103	0.058	0.171	0.113	7825.52
**CK2**	0.101	0.070	0.109	0.039	2754.33
**CK3**	0.105	0.156	0.217	0.061	4143.93

**T1-1**	0.104	0.158	0.277	0.119	5606.73
**T1-2**	0.103	0.172	0.281	0.109	5185.44
**T1-3**	0.101	0.126	0.327	0.201	9751.49
**T2-1**	0.105	0.163	0.356	0.193	9006.67
**T2-2**	0.101	0.146	0.338	0.192	9314.85
**T2-3**	0.104	0.098	0.361	0.263	12391.35
**T3-1**	0.104	0.042	0.338	0.296	13946.15
**T3-2**	0.104	0.036	0.335	0.299	14087.50
**T3-3**	0.102	0.047	0.346	0.299	14363.73
**T4-1**	0.102	0.024	0.196	0.172	8262.75
**T4-2**	0.104	0.052	0.251	0.199	9375.96
**T4-3**	0.101	0.14	0.241	0.101	4900.00
**T5-1**	0.1	0.096	0.222	0.126	6174.00
**T5-2**	0.1	0.103	0.204	0.101	4949.00
**T5-3**	0.1	0.062	0.253	0.191	9359.00

Table S7 SOD activity

**Sample**	**weight (g)**	**Measurement (560nm)**	**Control(560nm)**	**ΔAMeasurement**	**Percentage inhibition**	**Activity**
**CK1**	0.100	0.178	0.091	0.087	0.19	22.99
**CK2**	0.103	0.155	0.069	0.086	0.20	23.71
**CK3**	0.101	0.146	0.062	0.084	0.21	27.11

**T1-1**	0.1	0.129	0.061	0.068	0.36	57.35
**T1-2**	0.105	0.175	0.099	0.076	0.29	38.85
**T1-3**	0.103	0.141	0.068	0.073	0.32	45.22
**T2-1**	0.101	0.186	0.118	0.068	0.36	56.79
**T2-2**	0.101	0.188	0.124	0.064	0.40	66.52
**T2-3**	0.105	0.189	0.126	0.063	0.41	66.52
**T3-1**	0.102	0.176	0.126	0.05	0.53	111.76
**T3-2**	0.105	0.145	0.092	0.053	0.50	97.04
**T3-3**	0.1	0.17	0.118	0.052	0.51	105.77
**T4-1**	0.102	0.151	0.093	0.058	0.46	82.83
**T4-2**	0.1	0.121	0.062	0.059	0.45	81.36
**T4-3**	0.105	0.14	0.081	0.059	0.45	77.48
**T5-1**	0.103	0.172	0.108	0.064	0.40	65.23
**T5-2**	0.104	0.151	0.086	0.065	0.39	62.13
**T5-3**	0.1	0.195	0.131	0.064	0.40	67.19
